# Inhibition of tomato (*Solanum lycopersicum* L.) root growth by cyanamide is due to altered cell division, phytohormone balance and expansin gene expression

**DOI:** 10.1007/s00425-012-1722-y

**Published:** 2012-07-31

**Authors:** Dorota Soltys, Anna Rudzińska-Langwald, Agnieszka Gniazdowska, Anita Wiśniewska, Renata Bogatek

**Affiliations:** 1 Department of Plant Physiology, Warsaw University of Life Sciences-SGGW, Nowoursynowska 159, 02-776 Warsaw, Poland; 2 Department of Botany, Warsaw University of Life Sciences-SGGW, Nowoursynowska 159, 02-776 Warsaw, Poland; 3 Present Address: Laboratory of Biotechnology, Plant Breeding and Acclimatization Institute, National Research Institute, Research Division at Młochów, Platanowa 19, 05-831 Młochów, Poland

**Keywords:** Allelopathy, Auxin, Ethylene, Mitosis, Phytotoxicity

## Abstract

Cyanamide (CA) has been reported as a natural compound produced by hairy vetch (*Vicia villosa* Roth.) and it was shown also to be an allelochemical, responsible for strong allelopathic potential in this species. CA phytotoxicity has been demonstrated on various plant species, but to date little is known about its mode of action at cellular level. Treatment of tomato (*Solanum lycopersicum* L.) roots with CA (1.2 mM) resulted in inhibition of growth accompanied by alterations in cell division, and imbalance of plant hormone (ethylene and auxin) homeostasis. Moreover, the phytotoxic effect of CA was also manifested by modifications in expansin gene expression, especially in expansins responsible for cell wall remodeling after the cytokinesis (*LeEXPA9, LeEXPA18*). Based on these results the phytotoxic activity of CA on growth of roots of tomato seedlings is likely due to alterations associated with cell division.

**Electronic supplementary material:**

The online version of this article (doi:10.1007/s00425-012-1722-y) contains supplementary material, which is available to authorized users.

## Introduction

Cyanamide (CA), bioactive secondary plant product was first identified in hairy vetch (*Vicia villosa* subs. *varia* Roth) (Kamo et al. 2003). This leguminous plant is widely used as a winter cover crop for prevention of water evaporation and as a source of nitrogen when used as a green manure. However, hairy vetch inhibits the growth of various weed species, e.g. white star (*Ipomoea lacunosa* L.), barnyard grass (*Echinochloa crus*-*galli* L.), prickly sida (*Sida spinosa* L.) and hairy crabgrass (*Digitaria sanguinalis* L.) (Hoffman et al. 1993; Fujii 2001; Reddy and Koger 2004), and is used for weed suppression in Japanese crop production. Identification of phytotoxic compounds synthesized in hairy vetch seedlings pointed to CA as the key component. Cyanamide is present in all organs of hairy vetch, including seeds (inside endosperm), but is most abundant in plant shoots (approx. 444 mg kg^−1^ FW) (Kamo et al. 2003). Apart from hairy vetch, CA was also isolated from bird vetch (*V. cracca* L.) and black locust (*Robinia pseudoacacia* L.) (Kamo et al. 2006, 2008).

Nowadays, the term “phytotoxicity” is used to distinguish allelopathy (as phenomenon occurring in natural environment), from laboratory studies on phytotoxic compounds. Therefore, phytotoxicity refers to allelopathic interactions studied under controlled laboratory conditions, using plant extracts, phytochemicals isolated from plant tissue, collected from exudates or even synthetic compounds—identical to natural ones (Macias et al. 2003). Controlled laboratory experiments are design to reduce the impact of other biotic and abiotic factors.

One of the first visible effect of action of phytotoxins is restriction in seed germination or/and seedlings growth (for review see Gniazdowska and Bogatek 2005). Unfortunately, most studies describe only the visual symptoms or morphological impacts of phytotoxins, without detailed analysis of their physiological or biochemical mode of action. It should be noted that delay of seed germination or plant growth by phytotoxins, is often the final result of specific inhibition of key enzyme sites. Moreover, it should be pointed out, that mode of action of the specific phytotoxin in each plant organ (root, hycopotyl, leaves) may be different. Additionally, as described earlier most phytotoxins may be characterized by multidirectional mode of action (Gniazdowska and Bogatek 2005). Cinnamic acid, one of the phenolic compounds, induces membrane depolarization and inhibits absorption of some ions, e.g. phosphate, potassium, nitrate, and magnesium (Einhelling 2004). Moreover, it stimulates ROS generation, lipid peroxidation and inhibits catalase and peroxidase activity. Cinnamic acid alters also auxin biosynthesis and modifies photosynthesis and respiration rate (Einhelling 2004).

Cell division and cell elongation in seedling root are essential processes responsible for root growth. Cell division provides new cells that subsequently reside within the elongation zone, where cell enlargement occurs (Cosgrove 1997). Inhibition of mitosis leads to reduced root length, even without any effects on cell enlargement. A variety of phytotoxins act as inhibitors of division of root tip cells (Ding et al. 2008; Sánchez-Moreiras et al. 2008). Both physiological processes, mentioned above are controlled by phytohormones such as auxins and ethylene (Etheridge et al. 2005; Cho et al. 2007). A distinctive hormonal balance is required for undisturbed root growth. It is known that ethylene has the ability to inhibit root growth, but ethylene-regulated root growth is dependent on auxin transport. It was noted that alterations in any component of auxin transport and signal transduction result in impacts upon ethylene synthesis and signaling pathway and vice versa (Swarup et al. 2007). Briefly, increasing auxin concentration results in local ethylene precursor 1-aminocyclopropane-1-carboxylic acid (ACC) biosynthesis in root tip and ethylene diffusion and accumulation in elongation zone. Moreover, increasing ethylene concentration inhibits polar auxin transport in the root, resulting in its accumulation in the elongation zone (Smalle and Van Der Straeten 1997). Enhanced concentration of auxins and ethylene emission in the elongation zone results in inhibition of cell growth. However, cell enlargement in roots may not only be affected by altered plant hormone homeostasis, but may also be due to alterations in cell wall flexibility. Expansins are proteins that influence physical properties of cell walls (Cosgrove 2000) by cutting hydrogen bonds between cellulose and hemicellulose. Expansins also take part in cell wall remodeling after cytokinesis (Reinhardt et al. 1998; Vogler et al. 2003).

The objective of the present study was to investigate the physiological effects of CA on tomato (*Solanum lycopersicum* L.) root growth. The root is of special interest as it is a primary organ exposed to direct influence of potential phytotoxins that may be found in the soil. Our preliminary studies indicated that tomato may be used as an excellent model to investigate the mode of action of CA mostly due to its convenience in cytological observation and well known sequences of expansin genes. Moreover, previously we demonstrated that CA inhibited growth of onion (*Allium cepa* L.) roots in a dose-dependent manner (Soltys et al. 2011). Experiments conducted on tomato showed that CA-induced restriction in root growth was concentration (0.8–2 mM) dependent (Sołtys et al. 2010). Therefore, in our investigations we have chosen 1.2 mM CA concentration, as that one required for 50 % inhibition of root growth. We performed experiments to study two general aspects of root growth: cell division in root tips and cell enlargement in the elongation zone. Based on experimental findings, we hypothesize that inhibition of tomato root growth by CA is mainly due to disturbance of cell division in root tips as well as imbalances in phytohormone levels in developing roots, which result in altered expansin gene expression.

## Materials and methods

### Plant material

Tomato (*Solanum lycopersicum* L. cv. Malinowy Ożarowski) seeds were obtained from Horticultural Seed Company and Nursery “PNOS Ożarów” Ożarów Mazowiecki, Poland. Seeds germinated in water in darkness at 20 °C for 36 h. After radical protrusion (day 0) seedlings of equal size (5 mm root length) were transferred to distilled water (control) or aqueous CA-containing solution (0.8, 1.2, 2 mM). Culture of tomato seedlings was carried out in Petri dishes (ø 18 cm, 30 seedlings per dish) for 7 days in darkness at 20 °C. Length of seedling roots was determined after 7 days. In each of 5 replications, 40 seedling roots were measured.

### Recovery effect after CA treatment

After 1 or 3 days of CA (1.2 mM) treatment, seedlings were transferred into water-moistened Petri dishes containing saturated filter paper. Length of seedling roots was measured after additional 5 days recovery following treatment. Data were expressed as % of control (non-treated) plants. Experimental treatments were replicated four times and in each experiment, 25 roots were measured.

### Mitotic index

The mitotic index was determined using the carbol fuchsin staining method. After 1 or 3 days of CA (1.2 mM) treatment, distal fragments of roots (1 cm long) were cut off and fixed in Carnoy’s fixative (glacial acetic acid : ethanol, 3:1, w/v) for 24 h. Then, roots were washed three times for 15 min in 70 % ethanol and hydrolyzed in 1 M HCl at 60 °C, stained with carbol fuchsin (0.3 % basic fuchsin, 5 % phenol in distilled water, w/v) and transferred onto the slide. Root tips (2 mm long) were cut off and immersed in a drop of water. Frequencies of each mitotic phase were calculated by examining 1,000 cells. Results were expressed as %. The mitotic index was observed under light microscope Olympus AX70 PROVIS using programs View Finder and Studio Lite (Olympus).

### Length of root cells

After 3 days of culture in water or 1.2 mM CA solution, 8-mm-long segments of tomato roots (including root tip) were cut off and fixed in Karnovsky fixative (5 % glutaraldehyde, 4 % paraformaldehyde and 0.2 M sodium cacodylate, v/v) for 4 h at 4 °C. After rinsing in cacodylate buffer (0.2 M sodium cacodylate) root segments were dehydrated in an ethanol series (10, 20, 30, 40, 50, 60, 70, 80, 90 % for 10 min each) and embedded in epon resin 812 (Fluka) with propylene oxide in proportion 0:1, 1:3, 1:1 and 3:1 and left for propylene oxide evaporation. The embedded roots were separated into four (1–4) 2-mm-long segments and poured with epon resin and polymerized for 24 h at 60 °C. The samples were sectioned longitudinally and 3-μm-thick sections were transferred to slides. Each 2-mm-long root segments were conventionally divided into 3 or 4 sections. Length of cells in each section was measured. Photographs of root segments were provided using an Olympus AX70 PROVIS microscope with the programs View Finder and Studio Lite (Olympus). Length of cells was counted using the AnalySIS (SIS) program. Experiments were repeated two times with five replications each.

### Concentration of IAA

Indole-3-acetic acid (IAA) was determined by competitive enzyme-linked immunosorbent assay (ELISA) modified after Weiler et al. (1981). Freshly cut roots (0.2 g) from 0, 1, 2 and 3-days-old control and CA (1.2 mM) treated seedlings were homogenized in liquid nitrogen. Extraction of IAA was done according to Marcussen et al. (1988), using 80 % ethanol with butyl-hydroxy-toluene as antioxidant. The obtained extract was centrifuged at 15,000*g* for 15 min at 4 °C. The supernatant was left for total ethanol evaporation and then was suspended in TBS buffer (50 mM Tris, 150 mM NaCl, 1 mM MgCl_2_). Probes were methylated using diazomethane and diluted in PBS buffer (0.8 % NaCl, 0.02 % KCl, 0.02 % K_2_HPO_4_, 8.1 mM Na_2_HPO_4_). ELISA plates were filled with rat anti-mouse antibodies (25 μg ml^−1^) and incubated for 18 h at 4 °C. Next plates were rinsed with distilled water, and IAA extracts (50 μl) were added for each well and incubated for 1 h at 4 °C. After the incubation time, 50 μl of tracer (IAA conjugated with alkaline phosphatase, Agrisera) diluted 1:1,650 with TBS-G buffer (0.1 % gelatin in TBS buffer) was added to each well and incubated 3 h at 4 °C, then rinsed with distilled water. For enzymatic reaction, substrate for alkaline phosphatase, *p*-nitrophenol sodium phosphate (Sigma) was added and incubated for 1 h at 37 °C. IAA concentration was measured at 405 nm with referential wave 605 nm (Dynatech MR 5000). IAA determination was performed in three biological and four technical repetitions.

### Ethylene emission

Roots of 0, 1, 2, 3-days-old seedlings cultured in water or 1.2 mM CA solution were cut off (about 0.5 g), placed in 15 ml glass flask with 0.2 ml of water or 1 mM 1-aminocyclopropano-1-carboxylic acid (ACC) and left for 20 min. Then flasks were tightly closed and roots were incubated for 3 h at 30 °C. Ethylene content in the gas phase (1 cm^3^) was detected using gas chromatograph 330 Girdel France (Supelco-HayeSep 80/100 column, FID detector, temperature of injection/detection 120/150 °C). Histograms were analyzed using the HP CORE Chem Station computer program. The actual (-ACC) ethylene emission was detected without exogenous ACC, the potential ethylene emission (+ACC) was measured in the presence of 1 mM ACC. For each experiment three biological and four technical repetitions were performed.

### Expansin genes expression

Total RNA was isolated from roots of 0, 1, 2, 3-days-old seedlings grown in water or 1.2 mM CA solution. The samples were homogenized in liquid nitrogen and RNA was isolated according to the modified Chomczyński method (Rybka et al. 2009). Specific primers for expansin genes were used according to Fudali et al. (2008) and validated using Primer 3 release 1.1.0 program. Reverse transcription (RT) was made using RevertAid™ kit (Fermentas) according to manufacturer’s instruction. The RT reaction mixture (10 μl) consisted of 1× RT buffer, 1 mM of dNTP mix, 5 μM of random primers (oligo(dt)primer), 10 U of RNasin, 10 U reverse transcriptase and 0.02 μg total RNA. The cDNA was used for several polymerase chain reactions (PCR) with specific expansin primers: *LeEXPA1, LeEXPA2, LeEXPA4, LeEXPA5, LeEXPA8, LeEXPA9* or *LeEXPA18* (Fudali et al. 2008). The PCR mixture (10 μl) contained 1× Tag buffer, MgCl_2_ (1.75 mM), 0.2 mM of each dNTP, primers (0.25 μM each), Taq polymerase (0.01 U, Fermentas), cDNA as a negative control (water was applied in this step). For each expansin primer specific PCR conditions and number of cycles (30) were adjusted. As a positive control, PCR with 18S rRNA primers was performed for each repetition. The PCR products were checked using electrophoresis in 1.5 % agarose/TBE gels (100 mM Tris–HCl, 83 mM boric acid, 1 mM EDTA, pH 8.4) containing 0.5 μg ml^−1^ ethidium bromide (Midi Horizontal Electrophoresis Unit Set, Sigma). All reactions were performed using GeneAmp® PCR System 9700 cycler (Applied Biosystems) and visualized using transilluminator UV (Gel Logic 200, Molecular Imaging System). The relative transcript level was counted in relation to control using Kodak Molecular Imaging Software. For each expansin gene analysis, two biological and four technical repetitions were performed.

### Statistics

Data were analyzed using the STATISTICA 9.1 software. Normality of the distribution and homogeneity of variance was checked using Kolmogorov–Smirnov and Levene’s test, respectively. All showed normal distribution and similar variances, and they fulfilled conditions for use ANOVA. However, due to high sample size, mean values were computed and mean differences were calculated using Tukey’s studentized range test in the following experiments: length of roots, length of roots after recovery phenomenon and length of cells in each root zone. Standard error (SE) and coefficient intervals (CI) were also provided to indicate the variations associated with the particular mean values.

## Results

### CA inhibits tomato root growth

**Fig. 1:**
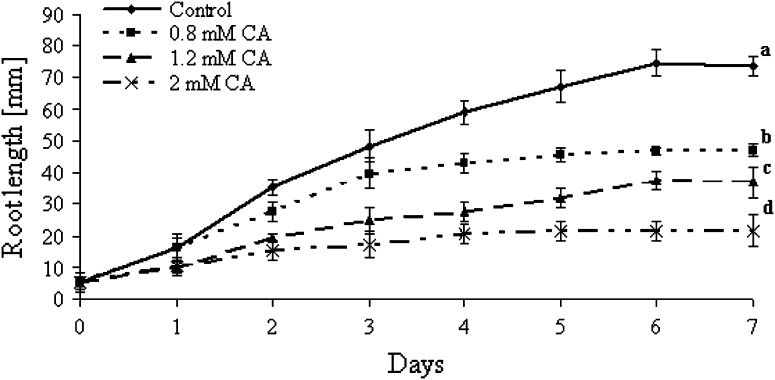
Length of roots of tomato seedlings cultured 7 days in water or CA solution at various concentration (0.8–2 mM) (mean ± SE, *n* = 200). *Different letters* indicate significant differences by Tukey’s test (*P* < 0.05)

**Fig. 2:**
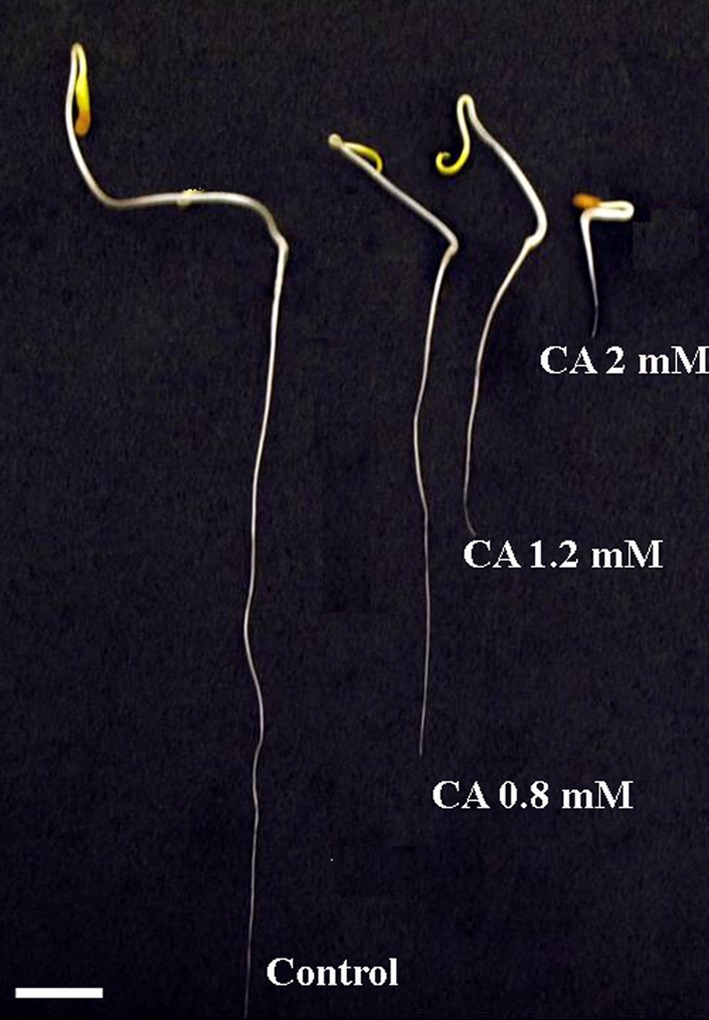
Tomato seedlings grown 7 days in water (*control*) or CA aqueous solutions (0.8; 1.2 and 2 mM). *Scale bar* 4.5 cm

Roots of control (untreated) tomato seedling continued growth during the entire culture period (Fig. 1). After 7 days the length of roots increased from 5 to 72 mm. Roots of seedlings treated with CA were much shorter than control roots. The inhibitory effect of CA treatment was dose dependent (Fig. 2). At the end of the culture period, roots treated with 0.8 mM CA reached approximately 47, 35 mm with 1.2 mM CA, and were only 22 mm long after treatment with 2 mM CA. Morphological (visible) differences between control and CA-treated roots were noticed after 3 days of culture. Roots had brown discoloration in the zone between root and hypocotyl and were light-brown along the entire length. The inhibitory effect of 1.2 mM CA on root growth increased as the experiment progressed. Roots treated with CA for 3 days were 50 % shorter in comparison to the control (Fig. 1). Therefore, in further tests the impact of CA on tomato root growth was determined only using the solution of the allelochemical at 1.2 mM concentration. Elongation growth of CA-treated roots was arrested after 3 days, as a consequence root length of older seedlings did not differ significantly from length of roots exposed to CA for only 3 days (Fig. 1). Consequently, all other tests were conducted only during the 3 days following experimental initiation.

**Fig. 3:**
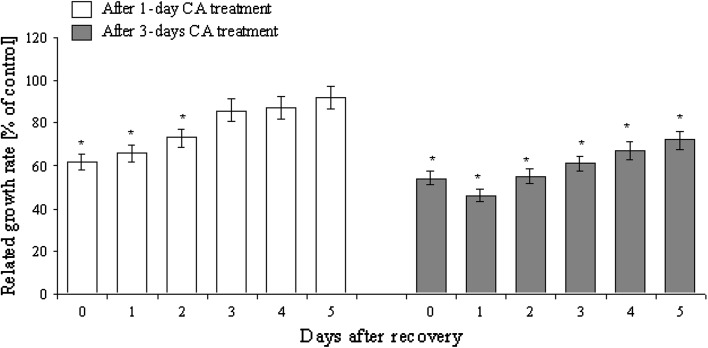
Length of roots (expressed in % of length of control) of tomato seedlings treated for 1 or 3 days with 1.2 mM CA and after 5 days of recovery in water (mean ± SE, *n* = 100). *Significant differences by Tukey’s test (*P* < 0.05)

The recovery phenomenon of CA (1.2 mM) treatment was time-dependent (Fig. 3). Effects of short-term (1 day) tomato seedling treatment with CA were fully reversible. After 5 days of recovery, roots reached 90 % of length of control plants (differences were statistically insignificant). Treatment with CA for 3 days led to irreversible alterations in root growth. After the first day of recovery, roots were nearly twofold shorter than the control. After additional 4 days root growth was detected, but treated roots did not reach the length of control roots (were 25 % shorter) (Fig. 3).

### CA modifies root tip cells division

The mitotic index in root tip cells of control seedlings was constant over the course of the experiment (Table 1). After 1, as well as 3 days of culture, percentage of dividing cells was unchanged and was 2.5 %. Similarly, no alterations in proportion of each phases of mitosis were detected in cells of CA (1.2 mM) treated root tips after short-term (1 day) exposure to phytotoxin. However, prolonged (3 days) CA treatment induced modifications in proportion of phases of mitosis in root tip cells. Two phases of mitosis: prophase and metaphase were inhibited in about 40 % in relation to control. This led to a decrease in percentage of dividing cells, from 2.60 to 1.74 % during 3 days of CA treatment (Table 1).

**Table 1:** Mitotic index in root tip cells of control and CA (1.2 mM) treated tomato seedlings after 1 or 3–days of culture (mean values ± SE, *n* = 10)

Phases of mitosis	Culture conditions	Dividing cells (%)	
		Days of culture	
		1	3
Prophase	Control	0.79 ± 0.12Aa	0.80 ± 0.10Aa
	CA	1.01 ± 0.12Aa	0.55 ± 0.10Bb
Metaphase	Control	0.82 ± 0.12Aa	0.81 ± 0.09Aa
	CA	0.74 ± 0.10Aa	0.50 ± 0.13Bb
Anaphase	Control	0.49 ± 0.10Aa	0.50 ± 0.08Aa
	CA	0.51 ± 0.06Aa	0.43 ± 0.11Aa
Telophase	Control	0.40 ± 0.04Aa	0.40 ± 0.05Aa
	CA	0.34 ± 0.05Aa	0.26 ± 0.08Bb
Cells in division (%)	Control	2.50 ± 0.28Aa	2.54 ± 0.23Aa
	CA	2.60 ± 0.21Aa	1.74 ± 0.36Bb

Frequencies of each mitotic phase were calculated for 1,000 cells. Results were expressed as %. Various statistical analyses were performed for each mitotic index component. Values for particular culture conditions followed by different capital letter (in rows) are significantly different at *P* < 0.05 (ANOVA test). Values for particular treatment (in columns) followed by different small letter are significantly different at *P* < 0.05 (ANOVA test)

### CA induces alterations in length of root segments

**Fig. 4:**
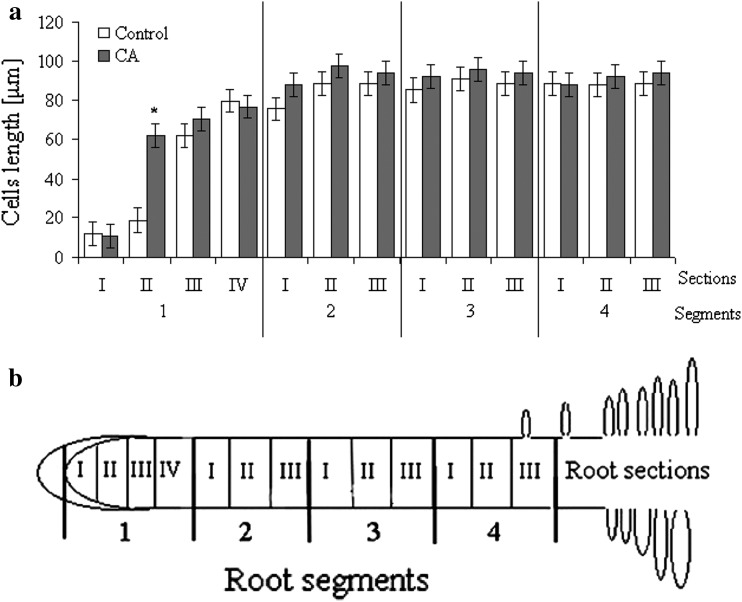
**a** Length of cells in various root segments of seedlings after 3 days of culture in water (*control*) or 1.2 mM CA solution (mean ± SE, *n* = 100). *Significant differences by Tukey’s test (*P* < 0.05). **b** Scheme of root divided into segments (1–4) and parts (I–IV) used for calculation of cell length

In control tomato roots the length of cells in distal segments (2–4) did not differ significantly. It was approximately 80 μm long in all sections of 2–4 segments (Fig. 4a). The greatest differences in cell length were detected in segment 1 (Fig. 4b). Cells in the two first apical parts (I and II) in segment 1 were less than 20 μm long. Cells in part III and IV of segment 1 were 62 and 80 μm long, respectively. Three-day-long treatment of tomato roots with 1.2 mM CA resulted in modification of cell length in part II of segment 1. Cells of this part of CA-treated roots were threefold longer as compared to control, and their length (approx. 60 μm) was similar to length of cells in part III, segment 1 of control roots. No significant differences in cell length of CA-treated and control plants were observed in other root segments.

### CA alters plant hormones homeostasis in roots

**Fig. 5:**
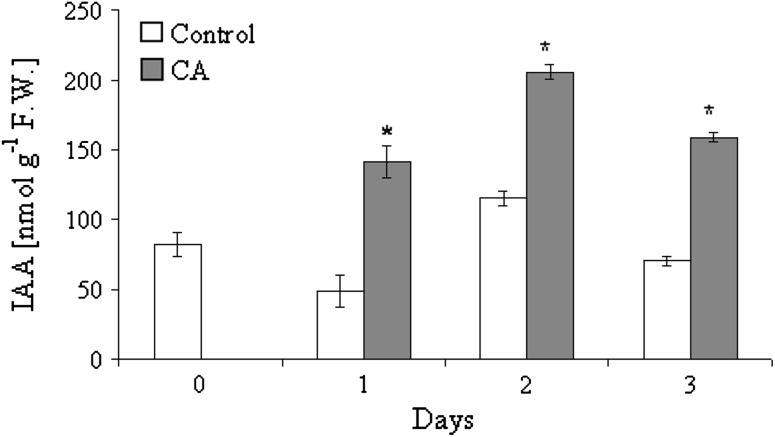
IAA concentration in roots of control and CA-treated tomato seedlings (mean ± SE, *n* = 12). *Significant differences by ANOVA test *P* < 0.05

Concentration of IAA in control roots fluctuated (rise and fall down) during 3 days of culture (Fig. 5) and varied from 45 to 110 nmol g^−1^ FW. Treatment with 1.2 mM CA significantly increased IAA concentration in roots, which was threefold higher than that in controls after 1 day, and almost doubled after 2–3 days (Fig. 5). The actual ethylene emission (−ACC) was lowest (0.13 μmol g^−1^ FW h^−1^) from control, untreated roots at the beginning of the experiment (Table 2). It increased almost sixfold (to the value 0.75 μmol g^−1^ FW h^−1^) within 1 day of culture and then decreased twice after additional 2 days. Fluctuation in ethylene emission from CA (1.2 mM) treated roots was similar to those observed in control plants, although rates of ethylene emission were significantly higher (for about 50 %) (Table 2). The highest ethylene emission from CA-treated roots was noted after 1 day of experiment, similarly as it was detected in control roots. Nevertheless, the most pronounced effect of CA on actual ethylene emission was observed after 3 days. Potential ethylene emission (+ACC) from control roots decreased continuously during culture period. The highest ethylene emission was noted at the beginning of the experiment, and then it declined sixfold at the end of the culture. CA increased the potential ethylene emission from roots of tomato seedling. After the first day of culture CA-treated roots emitted 36 % more ethylene than control ones. This effect was more pronounced in prolonged CA treatment and finally, potential ethylene emission from CA-treated root was 211 % higher than in control roots (Table 2). No ethylene emission was detected from pure CA solution and after incubation of CA with ACC (data not shown).

**Table 2:** Ethylene emission from root of control and CA-treated tomato seedlings (mean values ± SE, *n* = 12)

Days	Ethylene emission (μmol g^−1^ FW h^−1^)			
	−ACC		+ACC	
	Control	CA	Control	CA
0	0.13 ± 0.01	–	19.56 ± 1.66	–
1	0.75 ± 0.06a	0.98 ± 0.06b	10.50 ± 0.81a	14.34 ± 0.81b
2	0.27 ± 0.02a	0.34 ± 0.01b	4.29 ± 1.73a	12.01 ± 1.73b
3	0.31 ± 0.02a	0.48 ± 0.04b	3.14 ± 0.14a	9.76 ± 0.68b

Values for particular culture conditions (in rows, means ± CI) followed by different small letter are significantly different at *P* < 0.05 (ANOVA test)

### CA modifies expression of expansin gene

**Fig. 6:**
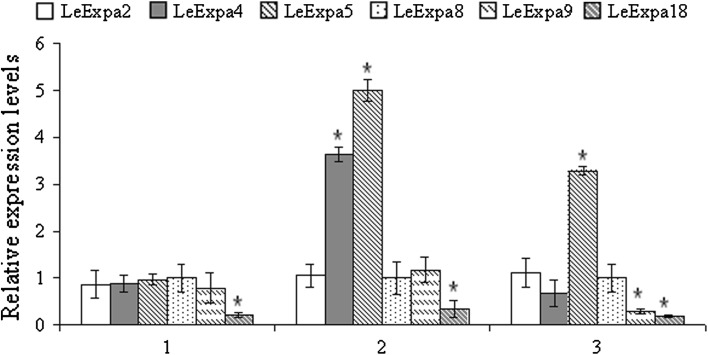
Relative transcript level of expansin gene. Value 1 means expression level equal to the control (mean ± SE, *n* = 8). *Significant differences by ANOVA test (*P* < 0.05)

The expression of the *LeEXPA* gene in control and CA-treated tomato seedlings during 3 days of culture is shown in Fig. 6. Among all analyzed genes, two (*LeEXPA2* and *LeEXPA8*) had unchangeable transcript abundance (bands of the same intensity) in all days of culture both in control and CA-treated plants (Fig. 6; Supplemental data Fig. S1). Expansins, *LeEXPA4* and *LeEXPA5* were found to be expressed at low level in control roots after 2 and 3 days of experiment, while in CA-treated roots *LeEXPA4* and *LeEXPA5* transcripts were still observed till the third day of culture. Expression pattern of some other expansin genes were also modified by 1.2 mM CA. Two of them (*LeEXPA*9 and *LeEXPA18*) were of special interest. Expression of *LeEXPA18* was strongly down-regulated in relation to control, already after 1 day of culture, while *LeEXPA9* after 3 days.

## Discussion

Many natural compounds derived from plant secondary metabolism are highly phytotoxic. It has been demonstrated that some natural products with high phytotoxic activity can induce severe alteration in plant growth due to induction of programmed cell death (PCD) (Ding et al. 2007; Keller et al. 2008) and restriction in cell division (Kuraś et al. 2006; Soltys et al. 2011; Teerarak et al. 2012).

We demonstrated that CA induced 50 % inhibition of root growth at quite high doses (1.2 mM), similarly as a lot of phytotoxins acting as plant growth inhibitors at high concentrations. Coumarin at a concentration of 1.3 mM affected onion root growth (Kupidlowska et al. 1994). Cineole, camphor and β-pinene (1 mM) inhibited turnip (*Brassica rapa* L. subsp. *campestris*) germination (Nishida et al. 2005). Benzoxazolin-2(3*H*)-one (BOA) led to 50 % inhibition of lettuce (*Lactuca sativa* L.) root growth at a concentration of 0.9 mM (Sánchez-Moreiras et al. 2008).

Our data show cellular alterations in the roots tip of tomato after treatment with CA (Fig. 4a). Moreover, we have shown that CA-induced inhibition of root growth was dose dependent (Fig. 1). This study validated also other data on CA phytotoxicity, e.g. treatment of lettuce with 10 μM CA inhibited root growth in about 40 % (Kamo et al. 2003), and onion roots exposed to CA 2 and 6 mM solutions were shortened in 30 and 60 %, respectively (Soltys et al. 2011). Similar restriction in root growth was also observed for other phytotoxins, e.g., cinnamic acid (Ding et al. 2007), chalcone (Díaz-Tielas et al. 2012), sorgoleone (Czarnota et al. 2003).

We investigated the reversible (recovery) effect of CA phytotoxicity after tomato seedlings treatment with CA and then post-incubation in pure water (Fig. 3). Three-day-long tomato culture in CA solution induced irreversible changes in root growth. Only roots treated with CA for 1 day reached the length of control roots after recovery, so we can conclude that prolonged exposition to CA provokes permanent perturbation in plant metabolism and structure. Moreover, we suspect that cellular mechanisms of CA detoxification were insufficiently expressed even after recovery. Unfortunately, recovery phenomenon after seedling treatment with phytotoxins is still poorly understood. However, data presented are in agreement with our previous studies on onion roots (Soltys et al. 2011). Only 1-day-long CA (2 mM) treatment of onion roots was fully reversible by bulbs transfer into distilled water (Soltys et al. 2011).

Inhibition of root growth by phytotoxins may be an effect of disturbances in cells division and differentiation. We demonstrated that CA may act as inhibitor of mitosis (Table 1). It decreased the percentage of dividing cells and frequencies of all phases of mitosis, apart from anaphases. The main reason for inhibition of mitosis after CA treatment is still not clear. Our former research showed that CA in higher concentration (10 mM) led to inhibition of mitosis in onion roots by disruption of the mitotic spindle organization and disturbances in chromosome condensation and organization in prophases and telophases (Soltys et al. 2011). However, it was also suggested that CA may arrest cell cycle in G2/M checkpoint (Soltys et al. 2011). Such an effect of other phytotoxins has been reported for water extract of cat’s claw (*Uncaria tomentosa* Willd.; 4 mg ml^−1^) which decreased the mitotic index in onion cells (Kuraś et al. 2006). Inhibition of cell division was observed in lettuce treated with BOA (1 mM; Sánchez-Moreiras et al. 2008), bean (*Pisum sativum* L.) exposed to sorgoleone (0.1 mM; Gattás-Hallak et al. 1999) or cockspur grass grown in extract of chinese perfume plant (*Aglaia odorata* Lour.; Teerarak et al. 2012). Leukamenin E induced disorder in chromosome morphology during mitosis in lettuce root tip cells (Ding et al. 2008).

In root tips, two types of cell proliferation occur: proliferative and quantal. In the first, after mitosis, daughter cells do not change their destination and still form the quiescent center, while cells under quantal cycle enter the differentiation zone (Gahan and Rana 1985). Inhibition of mitosis caused by CA decreases the number of cells under mitosis that may lead to shrinkage of the root tip, observed in our experiment (Fig. 4a). Root tips of CA-treated seedlings were approx. 50 % shorter than control ones. Altered root growth may also be an effect of disorder in cell differentiation in the elongation zone or in the transitional fragments of root tip and differentiation zones. In CA-treated roots dissimilarities/variations in cell length has been observed only in the first 2-mm-long segment, while in distal segments length of cells in control and treated roots remained equal. It suggests a shift in the root zones, but length of cells in part II of segment 1 is the same as in part III of segment 1. It might indicate that shortening of root tips correlates rather to earlier differentiation of cells after the end of mitosis, than to changes in cell length. Additionally, vacuolization, as the first sign of cell differentiation starts in plerom and periblem of treated roots earlier than in control ones (data not shown). Comparable effects have been observed in tobacco (*Nicotiana tabacum* L.) after treatment with cineol (440 μM; Yoshimura et al. 2011). Cineol influenced divisions in root tips, but did not affect the length of root cells. Moreover, monoterpenes (camphor, cineol, α-pinene and β-pinene) isolated from salvia (*Salvia leucophylla* L.) affected turnip root growth, although did not contribute to alterations in cells size (length and wide) (Nishida et al. 2005). On the other hand, inhibition of maize (*Zea mays* L.) root growth by hydroxycoumarin was due to nonsynchronized frequencies of cell division in vascular cylinder and cortex (Kupidlowska et al. 1994).

Cyanamide-dependent modification in root growth may be secondary effect of alterations in phytohormone balance. Both auxins and ethylene are responsible for correct root tip organization and root growth, e.g., for cell growth an inhibition of the local IAA concentration by ethylene is required (Swarup et al. 2007; Stepanova et al. 2007).

It is known that auxins are not sufficient, but important factors regulating cell division, differentiation and balance between the number of cells under mitosis and cells that enter differentiation, or post-division differentiation. Auxins control the cell cycle at early stages: at G1/S checkpoint and S phase before DNA replication starts. In our study after CA treatment in tomato roots an increased concentration of IAA and emission of ethylene was observed (Fig. 5; Table 2). Such variation in concentrations of phytohormones was detected in lettuce treated with leukamenin E and resulted in inhibition of root growth. Increasing IAA concentration led to overexpression of *WEI2/7*, the gene encoding antranilan synthase, an enzyme which catalyzes the synthesis of tryptophane—IAA’s precursor (Ding et al. 2008).

In our tests ethylene emission was higher in CA-treated roots. Measurement of ethylene emission with exogenous ACC relates to activity of ACC oxidase (ACO) that oxidized ACC into ethylene. Therefore, we assume that ACO activity is not affected by CA treatment and its activity is limited by substrate (ACC) availability. Moreover, it is possible, that ACO activity is stimulated by CA since the actual ethylene emission was enhanced by the phytotoxin. Our data are in agreement with the previously observed inhibition of *A. thaliana* cell growth in the root elongation zone, formation of root hairs and root thickening as a result of disorder in ethylene synthesis in (Le et al. 2001). It also confirms the involvement of ethylene in the plant reaction to phytotoxicity, since in oats (*Avena sativa* L.) the PCD has been induced after stimulation of ethylene emission by victorin (Yao et al. 2002).

Cells leaving root tip enlarge several doses due to increasing ploidy by endoreduplication and cell wall arrangement (Perrot-Rechenmann 2010). High turgor pressure is a main factor establishing cell wall reorganization and enlargement. Expansins are proteins that are mainly responsible for cell wall flexibility. They cut hydrogen bonds between cellulose and hemicelluloses molecules resulting in cell wall loosening. In tomato roots treated with CA, strong decrease of expression of *LeEXPA9* and *LeEXPA18* was observed (Fig. 6). Both of them (*LeEXPA9* and *LeEXPA18*) take part in cell expansion that accompanies cell division and are responsible for proper development of the root tip (Reinhardt et al. 1998; Vogler et al. 2003). It may indicate that the observed reduced value of the mitotic index in tomato roots could be a result of improper cell remodeling elicited by lower expression of expansins (*LeEXPA9* and *LeEXPA18*). On the other hand, the lack of significant changes in cell’s size can be a result of compensation by other expansin genes mainly responsible for cell enlargement, e.g. stable expression of *LeExpA2*, or even overexpression of *LeExpA4* and *LeExpA5* in roots treated with CA.

There is no other data about expansin gene expression and protein action under phytotoxic interaction, but their contribution in the regulation of development and growth of plant organs is well known. It was proven that the expression of *LeExpA2* occurs in different tomato root zones, hypocotyls, leaves and flowers, but not in meristematic tissues (Reinhardt et al. 1998; Vogler et al. 2003). Moreover, high correlation between transcript level of *LeExpA2* and hypocotyl growth was detected (Caderas et al. 2000). On the other hand, high expression of *LeExpa4* was shown in seeds (endosperm) of tomato already after 12 h of imbibition (Chen and Bradford 2000). Such data indicate its crucial role in cell wall loosening during seed coat disruption for radicle protrusion. Enhanced expression of expansins *LeExpA2 LeExpa4* and *LeExpA5* in plant organs characterized by high elongation ability may indicate that expansins play essential roles in the regulation of cells growth.

## Conclusions

All these results highlight the mode of action of CA as phytotoxin leading to inhibition of root growth, probably through the alteration in cell division, imbalance of plant hormones (ethylene and auxin) homeostasis and perhaps improper cell wall remodeling under cytokinesis by affecting expansin gene expression.

## Electronic supplementary material

Below is the link to the electronic supplementary material.

Supplementary material 1 (DOC 1.25 mb)

## Abbreviations

ACC: 1-Aminocyclopropane-1-carboxylic acid
ACO: 1-Aminocyclopropane-1-carboxylic acid oxidase
BOA: Benzoxazolin-2(3H)-one
CA: Cyanamide
DW: Dry weight
ELISA: Enzyme-linked immunosorbent assay
FW: Fresh weight
IAA: Indole-3-acetic acid
PCD: Programmed cell death
PCR: Polymerase chain reaction
RT: Reverse transcription
