# Supplementary material for: Inhibition of tomato (Solanum lycopersicum L.) root growth by cyanamide is due to altered cell division, phytohormone balance and expansin gene expression
Source: Planta. 2012 Jul 31;236(5):1629–38. doi: 10.1007/s00425-012-1722-y (PMC3481057; doi:10.1007/s00425-012-1722-y)
Supplement: Supplementary file 1 — Supplementary material 1 (DOC 1.25 mb) [file 425_2012_1722_MOESM1_ESM.doc]

**Inhibition of tomato (*Solanum lycopersicum* L.) root growth by cyanamide is due to altered cell division, phytohormone balance and expansin gene expression**

Dorota Soltys^1,3^, Anna Rudzińska-Langwald^2^, Agnieszka Gniazdowska^1^, Anita Wiśniewska^1^, Renata Bogatek^1^

*^1^Department of Plant Physiology, ^2^Department of Botany Warsaw University of Life Sciences-SGGW, Nowoursynowska 159, 02-776 Warsaw, Poland.*

*^3^ present address: Laboratory of Biotechnology, Plant Breeding and Acclimatization Institute - National Research Institute, Research Division at Młochów, Platanowa 19, 05-831 Młochów, Poland.*

Corresponding author: Agnieszka Gniazdowska [agnieszka_gniazdowska@sggw.pl](mailto:agnieszka_gniazdowska@sggw.pl), [gniazdowska@gmail.com](mailto:gniazdowska@gmail.com)

**Suplemental Figure S1**  RT-PCR analysis of the expression of expansin gene in roots of tomato seedlings grown in water (control) or 1.2 mM CA solution. 18S rRNA was used as control. Roots were collected after 0, 1, 2, and 3 days of culture.
